# Inflammatory markers associated with osteoarthritis after destabilization surgery in young mice with and without Receptor for Advanced Glycation End-products (RAGE)

**DOI:** 10.3389/fphys.2013.00121

**Published:** 2013-05-28

**Authors:** D. Justin Larkin, Jeffrey Z. Kartchner, Alexander S. Doxey, Weston R. Hollis, Jeffrey L. Rees, Spencer K. Wilhelm, Christian S. Draper, Danielle M. Peterson, Gregory G. Jackson, Chelsey Ingersoll, S. Scott Haynie, Elizabeth Chavez, Paul R. Reynolds, David L. Kooyman

**Affiliations:** Department of Physiology and Developmental Biology, Brigham Young UniversityProvo, UT, USA

**Keywords:** RAGE, TGF-β1, HtrA-1, Ddr-2, Mmp-13, osteoarthritis, OA, inflammation

## Abstract

HtrA1, Ddr-2, and Mmp-13 are reliable biomarkers for osteoarthritis (OA), yet the exact mechanism for the upregulation of HtrA-1 is unknown. Some have shown that chondrocyte hypertrophy is associated with early indicators of inflammation including TGF-β and the Receptor for Advanced Glycation End-products (RAGE). To examine the correlation of inflammation with the expression of biomarkers in OA, we performed right knee destabilization surgery on 4-week-old-wild type and RAGE knock-out (KO) mice. We assayed for HtrA-1, TGF-β1, Mmp-13, and Ddr-2 in articular cartilage at 3, 7, 14, and 28 days post-surgery by immunohistochemistry on left and right knee joints. RAGE KO and wild type mice both showed staining for key OA biomarkers. However, RAGE KO mice were significantly protected against OA compared to controls. We observed a difference in the total number of chondrocytes and percentage of chondrocytes staining positive for OA biomarkers between RAGE KO and control mice. The percentage of cells staining for OA biomarkers correlated with severity of cartilage degradation. Our results indicate that the absence of RAGE did protect against the development of advanced OA. We conclude that HtrA-1 plays a role in lowering TGF-β1 expression in the process of making articular cartilage vulnerable to damage associated with OA progression.

## Introduction

Osteoarthritis (OA) is one of the most common chronic diseases in the United States, and given the aging and obesity of the population, its prevalence is increasing. OA is characterized by joint pain, joint effusion, loss of mobility, and deformity that can progress to functional joint failure. More than 80% of people older than 65 years are symptomatic for OA (Chien et al., 2009).

Although the multifactorial nature of OA is well-recognized, little is known about the underlying molecular mechanisms leading to this disorder or the modalities that sustain them. OA is associated with several different inherited chondrodysplasia syndromes such as Stickler syndrome, caused by mutations in collagens or other cartilage specific genes. However, in most adults OA is associated with risk factors such as aging, obesity, repetitive stress, joint misalignment, acute injury, or genetic predisposition. Whether OA is triggered by mechanical stress or by a genetic abnormality, the end result is the same—a painful and progressive degeneration of the articular surface with thinning, fissuring, and ultimately loss of the functional, protective articular surface of the joint.

An important focus in the field of OA research at present is determining whether the different forms of OA that develop as a result of genetic predisposition, chronic joint stress, or acute injury all follow the same molecular pathway. We and others have shown that HtrA-1, Ddr-2, and Mmp-13, with HtrA-1 appearing first temporally, are components of a common molecular pathway (Murwantoko et al., 2004; Polur et al., 2010; Holt et al., 2012). OA progresses as HtrA-1 and Mmp-13, matrix-degrading enzymes, are synthesized and secreted from chondrocytes leading to the weakening of the articular cartilage. Early in the initiation of OA, an initial attempt to repair the region coincides with increased synthesis of cartilage matrix and increased proliferation of chondrocytes to form clones or clusters. Despite the attempt at early repair, the limited matrix synthesis is overwhelmed by the increased activity of matrix-degrading enzymes. The damaged cartilage surface, characteristic of late OA, is irreparable since adult articular chondrocytes cannot regenerate normal cartilage matrix. Importantly, this molecular metabolic pathway has also been previously demonstrated in surgically induced OA (Xu et al., 2010). Since HtrA-1, Ddr-2, and Mmp-13 are reliable biomarkers of OA, with HtrA-1 leading to the expression/upregulation of Ddr-2 and Mmp-13, a key to abrogating OA might lie in blocking the initiation of HtrA-1.

Formerly, OA was thought to be a disease of “wear and tear,” that is, a disorder in which mechanical forces physically degrade the articular cartilage surface. However, OA is increasingly viewed as a metabolically active, dynamic process involving inflammatory mediators, for review see Berenbaum (2013). In this scenario HtrA-1 upregulation may be associated with a biomarker(s) of an inflammatory pathway(s). It is, therefore, possible that blocking or inhibiting early stages of inflammation will attenuate OA.

The Receptor for Advanced Glycation End-products (RAGEs) are cell-surface receptors of the immunoglobulin superfamily expressed in diverse cell types. Studies have distinguished RAGE as a pattern recognition receptor that binds endogenous S100/calgranulins, amyloid-β-peptide, and HMGB-1 (or amphoterin), to influence gene expression via activated signal transduction pathways. Specifically, a host of pro-inflammatory responses such as those coordinated by MAP kinases, NF-κB, reactive oxygen species, and other pro-inflammatory mediators such as TNF, IL-1, TGF-β (Fukami et al., 2004) result from RAGE-ligand interactions. Engagement of RAGE by its ligands results in prolonged inflammation. Alarmins have been previously shown to be associated with OA, working through RAGE (Nakashima et al., 2012; Schelbergen et al., 2012). It is of interest to note that TGF-β1 has been shown to be involved both in chondrocyte regulation and the early pathogenesis of cartilage diseases (Grimaud et al., 2002).

In the present study, we examined the role of inflammation in OA by performing knee destabilization surgery on wild type and RAGE knock-out (KO) mice according to established procedures (Xu et al., 2010). We chose to use young mice to exclude any effect of aging on the development of OA. We report that OA was abrogated in RAGE KO mouse knee joints compared to destabilized joints from wild type mice. Furthermore, the data demonstrate an important relationship between RAGE, TGF-β1, and HtrA-1 in the progression of OA.

## Materials and methods

### Mice and joint destabilization procedure

RAGE KO mice that lack membrane and soluble RAGE were generated on a C57BL/6 background (Wendt et al., 2003). The RAGE KO have previously been shown to lack any RAGE expression (Wendt et al., 2003). Wild type/control mice were the same B57BL/6 background (The Jackson Laboratory, Bar Harbor, ME, USA; Strain Name: C57BL/6; Stock number 000664). The mice were randomized for sex and a destabilization of the medial meniscus (DMM) procedure was performed with 28-day-old-RAGE KO (*n* = 7) and wild type (*n* = 16) mice. Mice were anesthetized using an intraperitoneal injection of 0.5–0.7 cc Avertin IP [0.5 ml Avertin solution (15.5 ml tert-amyl alcohol to 25 g Avertin) in 39.5 ml normal saline solution] and the skin surrounding the right knee joint was prepped by clipping the fur and washing with an iodine surgical scrub followed by 70% alcohol. The remainder of the procedure was performed under a Wild Heerbrugg 355110 (Wild Heerbrugg AG, Switzerland) surgical microscope using sterile technique. The medial meniscotibial ligament was exposed by blunt dissection and the joint area was visualized. The meniscotibial ligament was subsequently transected using a number 11 scalpel to allow the displacement of the medial meniscus. Displacement of the meniscus was confirmed visually. The joint capsule and skin were both closed using 7-0 absorbable Vicryl suture (Ethicon, Inc., Somerville, NJ, USA). A sham procedure was also performed as a control on wild type (*n* = 5 for 28-day group, *n* = 3 for all other time points) and RAGE KO mice (*n* = 5) in which the meniscotibial ligament was exposed but not transected. These procedures were conducted under a protocol 10-0901 approved by the Brigham Young University IACUC.

### Tissue processing

Wild type mice at 3, 7, 14, and 28 days post-surgery (*n* = 3, 3, 3, and 7, respectively) and RAGE KO mice at 28 days post-surgery (*n* = 7) were euthanized by isoflurane USP (Abbott Laboratories, North Chicago, IL, USA), and right knee and temporomandibular (TM) joints were excised and fixed in 4% paraformaldehyde overnight. Each sample was then washed with H_2_O over a period of 6 h with the H_2_O being exchanged every 30 min. The tissues were decalcified in a formic acid solution that was changed every 2–3 days for a period of 2–3 weeks. Decalcification of each sample was confirmed through an ammonium oxalate reaction, and tissues were embedded in paraffin wax using an automated tissue processor (ThermoFisher Scientific, MA, USA). Paraffin blocks were prepared with the knee joint bent at a 120° angle, with the anterior tibial surface flush with the cutting surface. TM joints were embedded in paraffin wax with the joint flush with the cutting surface to achieve a frontal cut (Bomsta et al., 2006).

Right knee and TM joint blocks were sectioned at 6 μm through the entire joint from the anterior surface to the posterior using a Microm HM 325 microtome (Thermo Scientific, Kalamazoo, MI, USA). Four to five sections were placed per glass microscope slide, yielding ~35–50 slides or ~160–225 sections per joint, depending on the animal.

### Histological analysis

Histopathology was documented by Safranin O and Fast Green staining of every sixth slide of 3, 7, 14, and 28 days post-surgery knee samples. Using a light microscope equipped with an Olympus digital camera (Olympus America Inc., Center Valley, PA, USA), photographs of each knee joint were taken at 200 and 400× magnifications.

The articular cartilage in two representative sections from each stained slide was analyzed using a modified Mankin score to quantify the pathological state of each joint, with a score of 0 representing unaltered articular cartilage and 14 representing severe OA. Overall Mankin scoring was based on a subset of scores including cartilage erosion score (0–6), chondrocyte periphery staining (0–2), spatial arrangement of chondrocytes (0–3), and background staining intensity (0–3) (Mankin et al., 1971; Xu et al., 2003; Bomsta et al., 2006). Statistical significance of the combined Mankin scores for the 28 days RAGE KO and wild type surgery groups using a Two-Way ANOVA test conducted by the Department of Statistics at Brigham Young University.

### Immunohistochemical analysis

Immunohistochemistry was performed on sections of mouse knee joints from wild type (3, 7, 14, and 28 days post-surgery) and RAGE KO mice (28 days post-surgery). Separate slides were stained with antibodies against HtrA-1, TGF-β1, Ddr-2, and Mmp-13. Each slide was deparaffinized and then blocked for 1 h in PBS supplemented with 5% bovine serum albumin (Sigma-Aldrich, St Louis, MO, USA; B2518) and 0.05% Na Azide (Sigma-Aldrich, St Louis, MO, USA; S2002). Primary antibodies against HtrA-1 (ab38611) were purchased from Abcam (Cambridge, MA, USA), against TGF-β1 (ab92486) from Abcam (Cambridge, MA, USA), against Ddr-2 (SC-8989) from Santa Cruz Biotechnology (Santa Cruz, CA, USA), and against Mmp-13 (AB8120) from Chemicon (Temecula, CA, USA). All antibodies were diluted 1:200, applied to specimens, and incubated overnight at 4°C. On the second day, samples were rinsed with PBS and then incubated with an avidin/biotin ABC mix (Vectastain elite ABC Kit, Vector Laboratories, Inc., Burlingame, CA, USA). Slides were rinsed a second time with PBS and incubated with biotinylated secondary antibody. After a third PBS rinse, a color reaction was initiated to achieve a red/brown stain using a peroxidase substrate (VECTOR NovaRED substrate kit, Vector Laboratories, Burlingame, CA, USA). A cover slip was applied prior to photographing. Negative controls were prepared by staining without the addition of primary antibody. Differences in staining intensity were measured qualitatively with comparison to wild type control. The qualitative results of the immunohistochemical staining for the RAGE KO and wild type samples were analyzed statistically using Chi-Squared tests to evaluate differences between the two samples in staining patterns for key OA biomarkers. Similar statistical tests were used to also evaluate the relationship between the presence of TGF-β1 and HtrA-1 in RAGE KO and wild type samples. The relationship between immunohistochemical staining and severity of cartilage degradation was also evaluated using a Chi-Squared test. Staining was analyzed quantitatively by calculating the percentage of cells staining positive for the respective biomarkers and the total number of chondrocytes in a defined 200 × 900 pixel area of articular cartilage immediately distal to the tibial plateau. All quantitative analysis was performed using ImageJ (National Institutes of Health, Bethesda, MD, USA). Cell counting was done by two independent investigators who were blinded both to the strain of mouse (RAGE KO vs. wild type) and experimental group (sham vs. surgery). The quantitative results were subsequently analyzed statistically using an ANOVA test to detect differences in the mean percentages of positive staining for key OA biomarkers and mean chondrocyte counts between the RAGE KO and wild type samples.

## Results

### Rage KO mice are protected against OA

**Figure 1:**
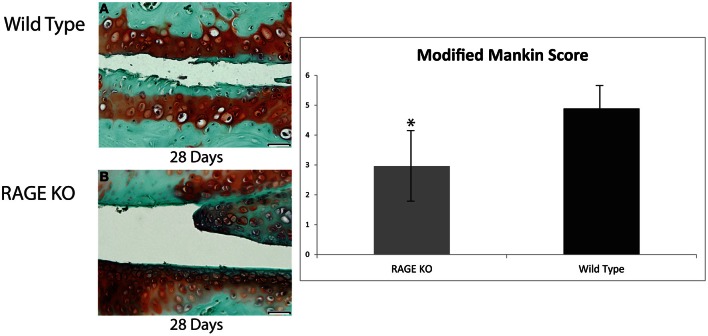
**Articular cartilage degradation from both wild type and RAGE KO samples was objectively evaluated using a modified Mankin score by two blind scorers.** Tissue samples were collected from Wild Type **(A)** and RAGE KO **(B)** at 28 days post-surgery and prepared with Safarnin O/Fast Green histological stains prior to Mankin scoring. Means of RAGE KO (*n* = 7) and wild type (*n* = 7) modified Mankin scores were compared to detect differences in cartilage degradation, and consequently, differences in OA. The mean of the wild type sample (modified Mankin Score = 4.89) was significantly higher than that of the RAGE KO sample (modified Mankin Score = 2.96) as determined by an ANOVA statistical test (^*^p < 0.05). The scale bar, valid for every image, is 50 μm.

Both RAGE KO and wild type mice were visualized and histologically analyzed (see Figure 1). While both RAGE KO and wild type mice demonstrated cartilage degradation following DMM surgery, tissue loss was markedly accelerated in the wild type mice. The cartilage degradation was indicated histologically by the early increase in proteoglycans, clustering of chondrocytes, and superficial fibrillation. In the RAGE KO mice, these indicators of OA were significantly attenuated. Wild type mice from the 3, 7, and 14 day groups demonstrated only early signs of cartilage degradation but a marked onset of OA was observed between the 14 and 28 days post-surgery. We consequently used day 28 as the time point to assess the effects of the surgery on OA.

To quantitatively evaluate the effect of RAGE on OA pathogenesis, the modified Mankin scores of the RAGE KO (*n* = 7) and wild type (*n* = 7) surgery and SHAM control mice (*n* = 3 for RAGE KO and wild type each) were evaluated 28 days post-surgery. Statistically significant differences were noted between the mean scores of the surgical and SHAM control groups (*p* < 0.01). More importantly, a statistically significant difference was also noted between the mean modified Mankin scores of RAGE KO (mean = 2.96) and wild type (mean = 4.89) mice at 28 days post-surgery (*p* < 0.05, see Figure 1). These results demonstrate that the absence of RAGE attenuates the progression of OA.

### Rage KO and wild type mice both show similar staining for key biomarkers

**Figure 2:**
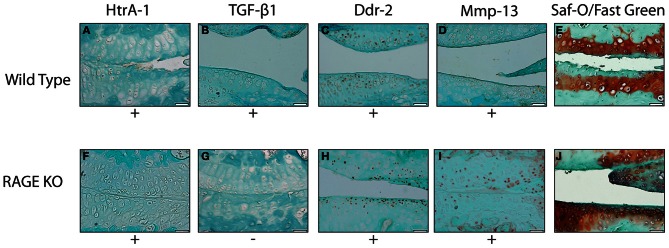
**Representative images showing the results of the immunohistochemical and histological stainings performed to analyze the presence of OA biomarkers and cartilage degradation.** All tissue samples were harvested 28 days after the DMM surgery. The images selected are representative of the trends observed within wild type **(A–E)** (*n* = 5) and RAGE KO **(F–J)** (*n* = 6) samples. The wild type and RAGE KO samples demonstrated similar staining patterns in all biomarkers except TGF-β 1 where the RAGE KO samples demonstrated fewer samples which stained positive. The difference was not statistically significant when tested with a Chi-Squared test (*p* < 0.35). The scale bar, valid for every image, is 50 μm.

Immunohistochemical analysis of the RAGE KO and wild type surgery groups 28 days post-surgery showed a positive staining for HtrA-1, Ddr-2, and Mmp-13 (See Figure 2). Wild type mice from the 3, 7, and 14 day groups demonstrated occasional, inconsistent staining for HtrA-1, Ddr-2, and Mmp-13. More tissue samples from the wild type sample stained positive for TGF-β1 than the RAGE KO sample, however no statistically significant was observed. Furthermore, statistical analysis revealed that there was no difference in the expression of HtrA-1, Ddr-2, and Mmp-13 when comparing the RAGE KO and wild type surgery groups (*p* = 0.30–0.65).

### Correlation between presence of TGF-β1 and HtrA-1

While our statistical analysis did not show any significant difference in the presence of TGF-β1 between RAGE KO and wild type mice (*p* < 0.35), we did observe a significant relationship between the presence of HtrA-1 and TGF-β1. We observed a pattern throughout the RAGE KO and wild type mice at 28 days post-surgery (see Table 1) such that when a tissue specimen stained positive for HtrA-1, it consistently stained negative for TGF-β1. Conversely, if the specimen stained negative for HtrA-1, it consistently stained positive for TGF-β1 (*p* < 0.01).

**Table 1:** **Illustrated here is the presence or absence of HtrA-1 and TGF-β1 in both wild type (WT) and RAGE KO samples as indicated by immunohistochemistry**.

**Mouse**	**HtrA-1**	**TGF-β1**
RAGE KO 1	+	−
RAGE KO 2	+	−
RAGE KO 3	+	−
RAGE KO 4	−	+
RAGE KO 5	+	−
WT 1	+	−
WT 2	+	−
WT 3^*^	+	+
WT 4	+	−
WT 5	−	+
WT 6	+	−

* Indicates the only exception to the observed trend.

A significant trend was observed between the presence of HtrA-1 and TGF-β1 in both the RAGE KO and wild type samples. With the exception of WT 3, all samples demonstrated a trend where samples which stained positive for HtrA-1 consistently stained negative for TGF-β1, while samples which stained negative for HtrA-1 consistently stained positive for TGF-β1. The statistical significance of this pattern was tested using a Chi-Squared test and was found to be highly significant (p < 0.01).

### Differences in total number of chondrocytes and percentage of chondrocytes staining positive for OA biomarkers

**Figure 3:**
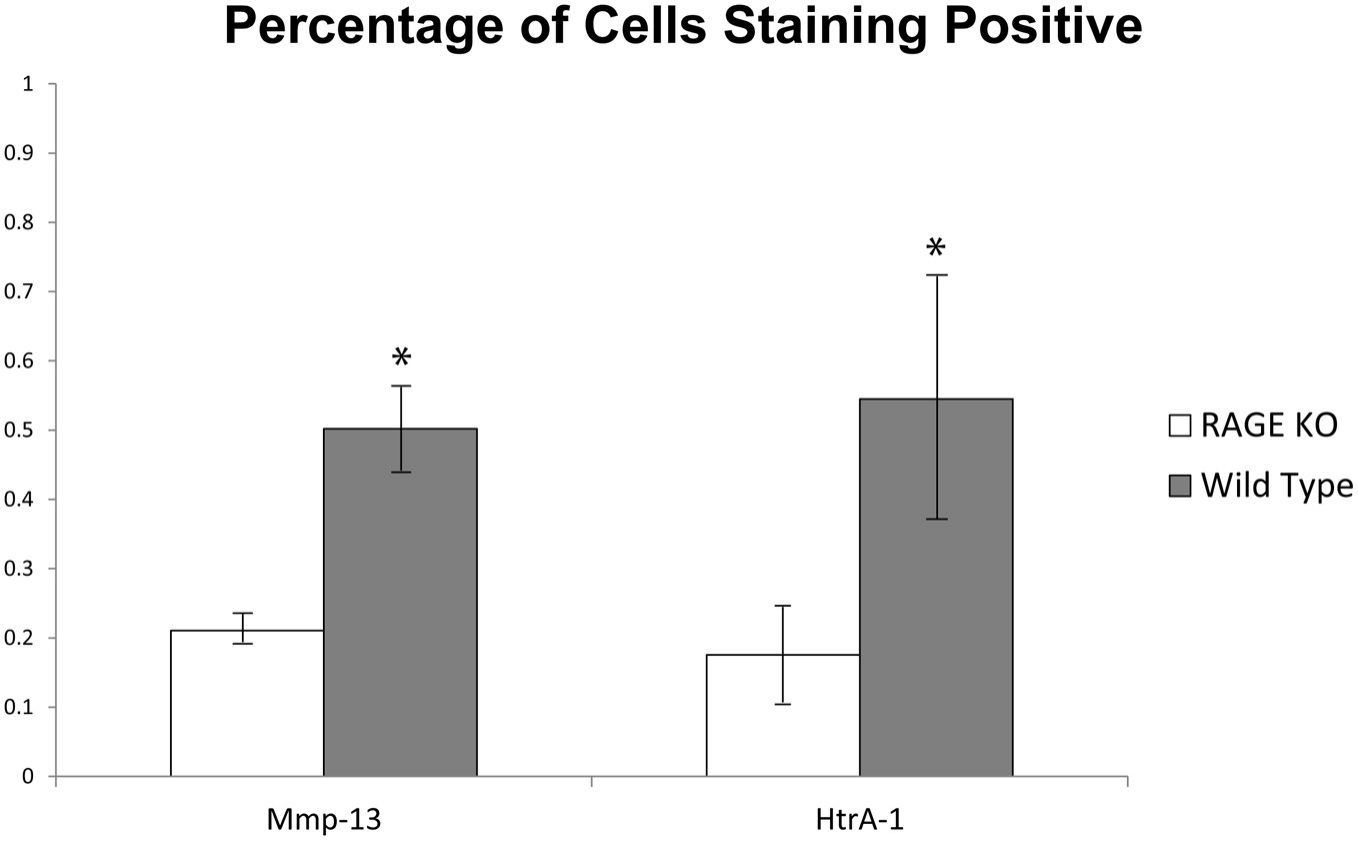
**To resolve the similar immunohistochemical staining patterns observed between RAGE KO and wild type samples despite the protective effect seen in RAGE KO joints, the percentage of cells staining positive for the given biomarkers was calculated at 400× magnification in a defined 200 × 900 pixel area of articular cartilage immediately distal to the tibial plateau.** While both RAGE KO (*n* = 5) and wild type (*n* = 6) samples stained positive for Mmp-13 and HtrA-1, the wild type mice had a significantly higher percentage of cells which stained positive for both Mmp-13 and Htr-A1 (^*^p < 0.03 and *p* < 0.02 respectively).

**Figure 4:**
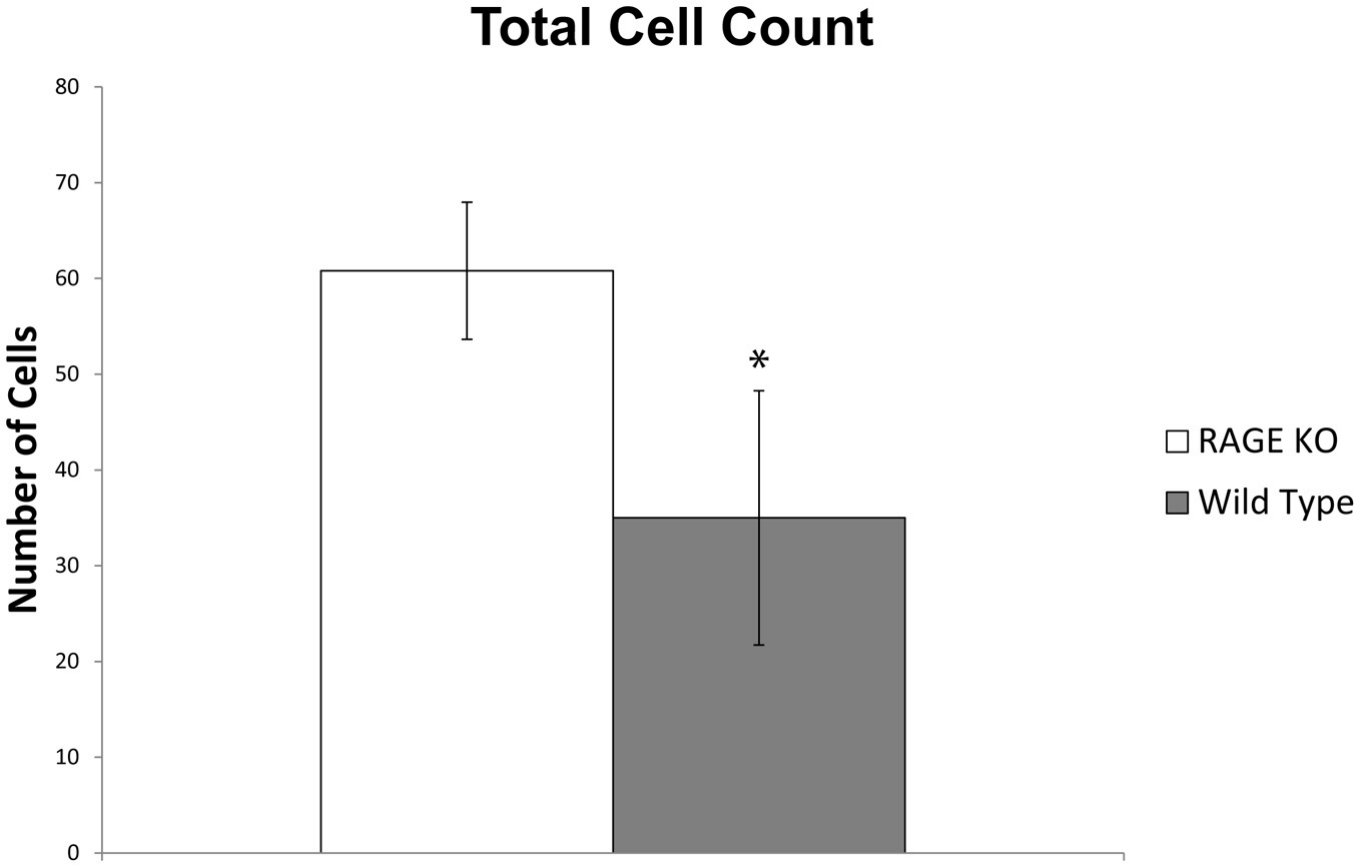
RAGE KO samples (*n* = 5), when compared to wild type samples (*n* = 6), demonstrated a significantly higher number of total chondrocytes counted in a defined 200 × 900 pixel area of articular cartilage immediately distal to the tibial plateau at 400× magnification (^*^p < 0.02).

A quantitative analysis of the immunohistochemical staining elucidated key differences in the percentage of chondrocytes staining positive for HtrA-1 and Mmp-13 in RAGE KO and wild type samples. RAGE KO tissue specimens, which had consistently less cartilage degradation, also showed a significantly lower percentage of chondrocytes, per defined area of articular cartilage, which stained positive for HtrA-1 and Mmp-13 when compared to wild type samples (*p* < 0.02 for HtrA-1 and *p* < 0.03 for Mmp-13, see Figure 3). Furthermore, RAGE KO samples also consistently demonstrated a higher number of chondrocytes in a defined area of articular cartilage (mean = 60.8) when compared to wild type samples (mean = 35, *p* < 0.02, see Figure 4).

### Immunohistochemical staining correlated with severity of cartilage degradation

**Figure 5:**
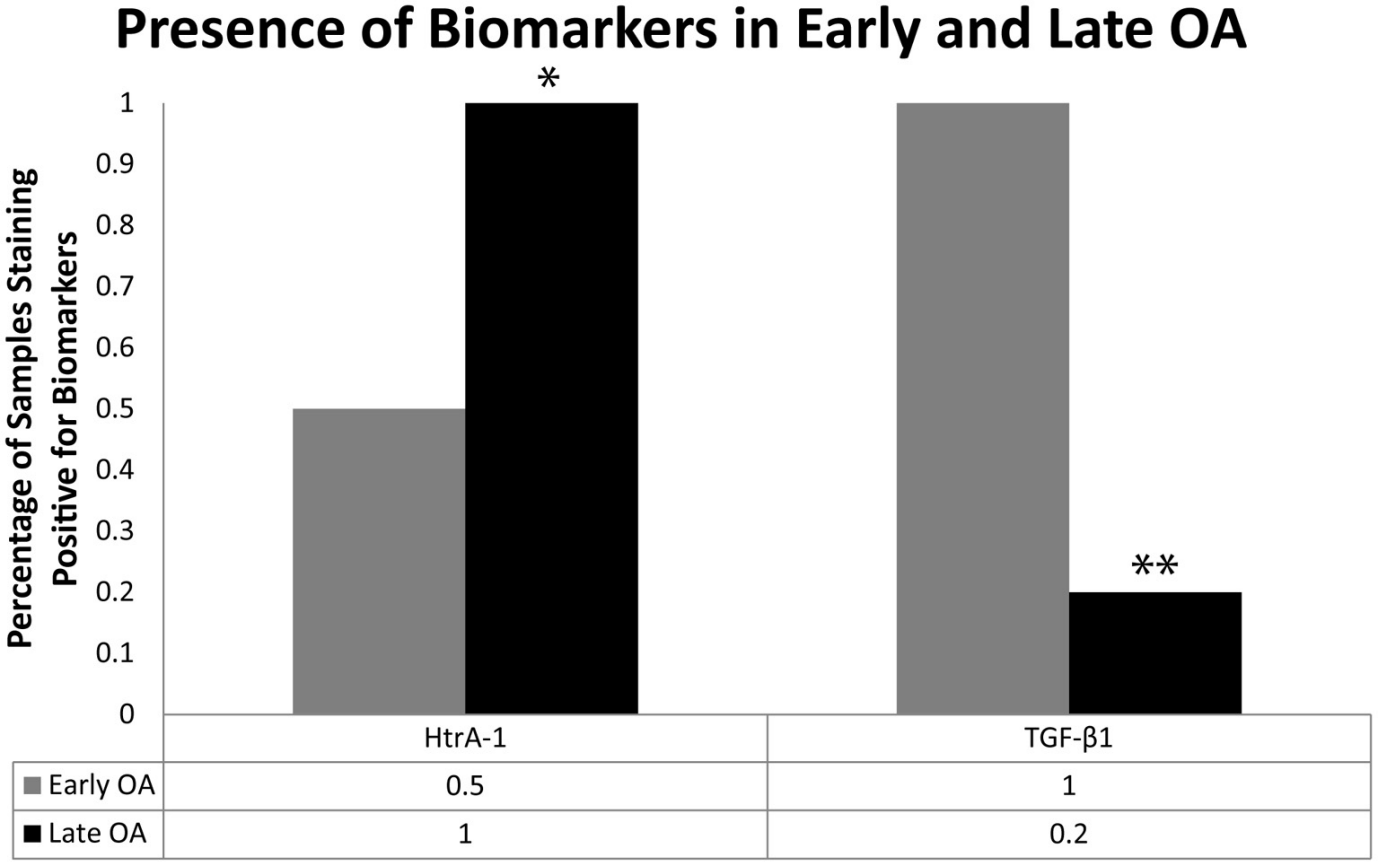
**Mice (*n* = 24) were sorted independent of mouse type (wild type vs. RAGE KO) and procedure (control vs. surgery) based on the severity of cartilage degradation as indicated by the modified Mankin score.** The frequency of positive immunohistochemistry staining for HtrA-1 and TGF-β1 for early OA (Modified Mankin score of 1.5–3, *n* = 6) and late OA (Modified Mankin score >4, *n* = 5) samples are shown here. The trend in frequency of positive staining was compared using a Chi-Squared test. Positive TGF-β1 was observed at a high frequency in early OA samples, but at a significantly lower frequency in late OA samples (^**^p < 0.01). Conversely, the frequency of HtrA-1 was significantly higher in late OA samples than in early OA samples (^*^p < 0.05).

When all mice samples from the 28 days post-surgery group (RAGE KO and wild type samples; surgery and sham control samples, *n* = 24) were combined and sorted by modified Mankin score, significant differences were observed in the presence of key OA biomarkers. Specifically, the presence of Ddr-2 and HtrA-1 was observed in every specimen categorized as late OA (Mankin scores >4, *n* = 8) in both the RAGE KO and wild type samples. In contrast, samples characterized as early OA (modified Mankin Scores of 1.5–3, *n* = 6), showed a lower frequency of positive staining for HtrA-1 (*p* < 0.05, see Figure 5). Positive staining for Mmp-13 was also consistently observed in mice specimen of both samples categorized as late OA.

When the RAGE KO and wild type samples were sorted by modified Mankin score, a difference (*p* < 0.01) was also observed for the presence of TGF-β1. In samples with minimal cartilage degradation (modified Mankin scores <1.5), TGF-β1 was absent in almost all samples (data not shown). At levels of cartilage degradation characterized as early OA, all samples stained positive for TGF-β1 (Figure 5). Finally, samples with cartilage degradation characterized as late OA, exhibited less staining for TGF-β1 (*p* < 0.01).

## Discussion

Effective treatment of OA is dependent on identifying the key biomarkers and signaling mechanisms involved in its pathogenesis. Generally, previous research has described OA as a process of mechanical degradation. More recently, it has suggested that inflammatory processes may be key players in the early onset of the disease (Berenbaum et al., 2013; Chockalingam et al., 2013; Berenbaum, 2013). In the present study, we have demonstrated clear involvement of RAGE and TGF-β1 in the pathogenesis and progression of OA.

Management strategies of articular cartilage injuries or defects in children and young adults are controversial and problematic (Micheli et al., 2006; Macmull et al., 2011; Schmal et al., 2013). Of consideration is determining the relative age of cartilage based upon chondrocyte-specific markers. HtrA1 has previously been shown to be important in regulating OA in humans (Hadfield et al., 2008; Tiaden et al., 2012). Our study utilized mice that were 4 weeks of age at initiation of surgery. Even at this early age we demonstrate that OA progression followed a similar metabolic pathway compared to what we have previously reported in older mice (Holt et al., 2012). This lends support to the idea that regardless of age or etiology, OA proceeds in a similar manner.

Our results indicate that the absence of RAGE in mice does provide a protective effect against the development of advanced OA. These results and previous work (Zreiqat et al., 2010) contradict the results of a study in which OA was induced by an ACL destabilization process (Cecil et al., 2009). We believe this discrepancy is caused by the severity of the ACL procedure and the consequent morphological joint changes that occur.

While our results demonstrate that RAGE is a key biomarker in the onset of OA, it is important to note that RAGE KO mice still experienced moderate levels of cartilage degradation following the DMM procedure. These results suggest that other secondary inflammatory signaling pathways closely associated with the RAGE pathway, such as those initiated by Toll-like Receptors, may also be activated following the surgical procedure (Bierhaus et al., 2005; Bianchi, 2007).

Even though the histological analysis of the RAGE KO mice showed less severe cartilage degradation as indicated by the modified Mankin score, RAGE KO samples still showed a high frequency of staining for OA biomarkers (HtrA-1, Ddr-2, and MMP-13). It is important to recognize that IHC is not a quantitative analysis. A quantitative assessment of immunohistochemical staining demonstrated a lower percentage of RAGE KO chondrocytes staining positive for these biomarkers as compared to wild type samples (Figure 3). Additionally, we observed a higher number of chondrocytes per given area in the articular cartilage. The combination of these factors suggest that RAGE KO mice have lower concentrations of the key OA biomarkers that mediate the progression and onset of the disease (HtrA-1, Ddr-2, and MMP-13) and that the tissue is healthier. Future research will be focused on evaluating why RAGE KO mice are able to sustain higher number of chondrocytes in the presence of mechanical stress. Our current hypothesis is that RAGE KO mice are experiencing less apoptosis and consequently less cartilage degradation leading to OA.

Our results on TGF-β1 and HtrA-1 suggest a relationship between these two biomarkers. Transforming growth factor β1 (TGF-β1) has recently been implicated as a biomarker of OA because of its role in the regulation of HtrA-1 in articular cartilage (Li et al., 2012). TGF-β1 primarily functions in the SMAD signaling pathway and is considered a multifunctional cytokine involved in many cellular processes including cell growth, proliferation, differentiation, apoptosis, and the elaboration of extracellular matrix. TGF-β1 has been implicated in cartilage repair (van Beuningen et al., 2000). While the exact interaction between RAGE and TGF-β1 is still unclear, it has been suggested that cross talk occurs between similar to mothers against decapentaplegic (SMAD) and RAGE-mediated p38 MAPK signaling pathways required for TGF-β1-induced apoptosis in epithelial cells (Dziembowska et al., 2007).

As reported in this present work, HtrA-1 positive staining was only observed in tissues which stained negative for TGF-β1. Furthermore, tissues with moderate cartilage degradation, early OA, consistently stained positive for TGF-β1, while it was essentially non-existent in specimen with late OA in which HtrA-1 was present. These observations are consistent with previous work demonstrating that HtrA-1 binds to and inhibits a broad range of TGF-β1 family proteins (Oka et al., 2004). The present work suggests that when HtrA-1 is expressed as OA progresses, TGF-β1 is inhibited. This could have significant implications in this disease. It is possible that in addition to degrading components of the ECM, HtrA-1 may also inhibit the control of the inflammatory response generated early in OA, serving to exacerbate the progression of the disease.

The combination of our findings and those reported by others (Oka et al., 2004) support the hypothesis that TGF-β1 is present during the early onset of OA, induces the expression of HtrA-1, and is then down-regulated, or potentially inhibited by HtrA-1. Accordingly, if TGF-β1 is responsible for inducing the expression of HtrA-1 at early stages of OA, it may serve as a valuable pharmaceutical target for future OA treatments. We are currently examining that scenario.

### Conflict of interest statement

The authors declare that the research was conducted in the absence of any commercial or financial relationships that could be construed as a potential conflict of interest.
